# Supplementary material for: Modeling glioblastoma heterogeneity as a dynamic network of cell states
Source: Mol Syst Biol. 2021 Sep 16;17(9):e10105. doi: 10.15252/msb.202010105 (PMC8444284; doi:10.15252/msb.202010105)
Supplement: Supplementary file 6 — Source Data for Figure 5 [file MSB-17-e10105-s004.zip › Figure5A_sourcedata/GSEA_3017/nosigdb_stateA.GseaPreranked.1622795026485/DOROSZKO_U3180MG_CLUSTER_5.html]

Details for gene set DOROSZKO\_U3180MG\_CLUSTER\_5[GSEA]

|  || Dataset | state53017 |
| Phenotype | NoPhenotypeAvailable |
| Upregulated in class | na\_neg |
| GeneSet | DOROSZKO\_U3180MG\_CLUSTER\_5 |
| Enrichment Score (ES) | -0.74167514 |
| Normalized Enrichment Score (NES) | -2.8229492 |
| Nominal p-value | 0.0 |
| FDR q-value | 0.0 |
| FWER p-Value | 0.0 |
Table: GSEA Results Summary

  

Fig 1: Enrichment plot: DOROSZKO\_U3180MG\_CLUSTER\_5      
 Profile of the Running ES Score & Positions of GeneSet Members on the Rank Ordered List

  

| PROBE | GENE SYMBOL | GENE\_TITLE | RANK IN GENE LIST | RANK METRIC SCORE | RUNNING ES | CORE ENRICHMENT || 1 | PLP1 |  |  | 272 | 0.338 | -0.2571 | No |
| 2 | MT1X |  |  | 285 | 0.331 | -0.2467 | No |
| 3 | IDI1 |  |  | 334 | 0.314 | -0.2745 | No |
| 4 | GAP43 |  |  | 362 | 0.306 | -0.2813 | No |
| 5 | EMP1 |  |  | 724 | -0.322 | -0.6313 | No |
| 6 | ID2 |  |  | 727 | -0.323 | -0.6111 | No |
| 7 | NRN1 |  |  | 740 | -0.336 | -0.6003 | No |
| 8 | SLC4A4 |  |  | 763 | -0.355 | -0.5985 | No |
| 9 | GJA1 |  |  | 823 | -0.405 | -0.6314 | No |
| 10 | ID1 |  |  | 931 | -0.651 | -0.6968 | Yes |
| 11 | CD44 |  |  | 957 | -0.851 | -0.6639 | Yes |
| 12 | CRYAB |  |  | 967 | -0.940 | -0.6084 | Yes |
| 13 | LGALS3 |  |  | 970 | -0.989 | -0.5423 | Yes |
| 14 | ANXA1 |  |  | 972 | -1.018 | -0.4731 | Yes |
| 15 | LPL |  |  | 980 | -1.387 | -0.3847 | Yes |
| 16 | ID3 |  |  | 981 | -1.505 | -0.2810 | Yes |
| 17 | CCL2 |  |  | 985 | -1.881 | -0.1544 | Yes |
| 18 | GFAP |  |  | 987 | -2.255 | -0.0000 | Yes |
Table: GSEA details [plain text format]

  

Fig 2: DOROSZKO\_U3180MG\_CLUSTER\_5: Random ES distribution      
 Gene set null distribution of ES for **DOROSZKO\_U3180MG\_CLUSTER\_5**
